# Measuring What Matters: Multidimensional Dementia Care Support Needs in Home Health

**DOI:** 10.1093/geroni/igaf122.4282

**Published:** 2025-12-31

**Authors:** Sara Knox, Janet Horn, Kit Simpson, Julie Bynum

**Affiliations:** Medical University of South Carolina, Charleston, South Carolina, United States; Medical University of South Carolina, Medical University of South Carolina, South Carolina, United States; Medical University of South Carolina, Medical University of South Carolina, South Carolina, United States; University of Michigan, Ann Arbor, Michigan, United States

## Abstract

Home health clinicians need scalable tools to assess the complex care needs of people living with dementia (PLWD). Existing approaches are multidimensional, burdensome, or not integrated into routine workflows. The objective of this study was to develop and validate a care needs scale for PLWD using items from the Outcomes Assessment Information Set (OASIS), establishing a foundation for future integration into documentation systems. We analyzed 2019 Research Identifiable Files (OASIS, HHA, MBSF, MedPAR) including 1,376,556 PLWD in the development cohort (60%) and 917,704 in the validation cohort (40%). Factor 1 (functional/ADL support) was analyzed separately due to its dominant variance (eigenvalue 12.18, ∼81% variance explained). Four additional factors were derived using exploratory factor analysis with orthogonal rotation. Factor loadings were high (0.70–0.95), communalities exceeded 0.70, and weighted beta scores produced individual-level summary scores. The five care support domains are: (1) Functional & ADL Support, (2) Behavioral/Psychiatric Support, (3) Memory & Decision-Making Assistance, (4) Hospitalization Risk, and (5) Challenging Behaviors & Aggression Management. Functional/ADL items showed broad variation (means 1.72–3.83, SD 1.63–2.30), demonstrating meaningful differentiation across patients. This validated, multidimensional scale leverages routinely collected clinical data to characterize PLWD care needs. Future work will evaluate integration into documentation systems and examine relationships with outcomes, offering a scalable tool to support research, clinical decision-making, and policy innovation in dementia care.

